# Increasing 28 mitogenomes of Ephemeroptera, Odonata and Plecoptera support the Chiastomyaria hypothesis with three different outgroup combinations

**DOI:** 10.7717/peerj.11402

**Published:** 2021-06-22

**Authors:** Dan-Na Yu, Pan-Pan Yu, Le-Ping Zhang, Kenneth B. Storey, Xin-Yan Gao, Jia-Yong Zhang

**Affiliations:** 1 Key Lab of Wildlife Biotechnology, Conservation and Utilization of Zhejiang Province, Zhejiang Normal University, Jinhua, Zhejiang, China; 2 The Department of Biology, College of Chemistry and Life Science, Zhejiang Normal University, Jinhua, Zhejiang, China; 3 Department of Biology, Carleton University, Ottawa, Canada

**Keywords:** Mitochondrial genome, Palaeoptera, Chiastomyaria, Metapterygota, Phylogeney, Gene rearrangement

## Abstract

**Background:**

The phylogenetic relationships of Odonata (dragonflies and damselflies) and Ephemeroptera (mayflies) remain unresolved. Different researchers have supported one of three hypotheses (Palaeoptera, Chiastomyaria or Metapterygota) based on data from different morphological characters and molecular markers, sometimes even re-assessing the same transcriptomes or mitochondrial genomes. The appropriate choice of outgroups and more taxon sampling is thought to eliminate artificial phylogenetic relationships and obtain an accurate phylogeny. Hence, in the current study, we sequenced 28 mt genomes from Ephemeroptera, Odonata and Plecoptera to further investigate phylogenetic relationships, the probability of each of the three hypotheses, and to examine mt gene arrangements in these species. We selected three different combinations of outgroups to analyze how outgroup choice affected the phylogenetic relationships of Odonata and Ephemeroptera.

**Methods:**

Mitochondrial genomes from 28 species of mayflies, dragonflies, damselflies and stoneflies were sequenced. We used Bayesian inference (BI) and Maximum likelihood (ML) analyses for each dataset to reconstruct an accurate phylogeny of these winged insect orders. The effect of outgroup choice was assessed by separate analyses using three outgroups combinations: (a) four bristletails and three silverfish as outgroups, (b) five bristletails and three silverfish as outgroups, or (c) five diplurans as outgroups.

**Results:**

Among these sequenced mitogenomes we found the gene arrangement *IMQM* in Heptageniidae (Ephemeroptera), and an inverted and translocated *tRNA-Ile* between the 12S RNA gene and the control region in Ephemerellidae (Ephemeroptera). The *IMQM* gene arrangement in Heptageniidae (Ephemeroptera) can be explained via the tandem-duplication and random loss model, and the transposition and inversion of *tRNA-Ile* genes in Ephemerellidae can be explained through the recombination and tandem duplication-random loss (TDRL) model. Our phylogenetic analysis strongly supported the Chiastomyaria hypothesis in three different outgroup combinations in BI analyses. The results also show that suitable outgroups are very important to determining phylogenetic relationships in the rapid evolution of insects especially among Ephemeroptera and Odonata. The mt genome is a suitable marker to investigate the phylogeny of inter-order and inter-family relationships of insects but outgroup choice is very important for deriving these relationships among winged insects. Hence, we must carefully choose the correct outgroup in order to discuss the relationships of Ephemeroptera and Odonata.

## Introduction

**Figure 1:**
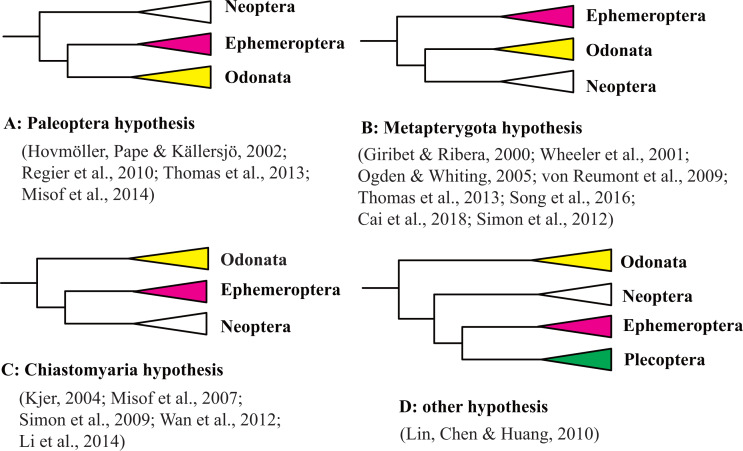
Three hypothesis of the relationships among Ephemeroptera, Odonata and Neoptera. (A) Paleoptera hypothesis; (B) Metapterygota hypothesis; (C) Chiastomyaria hypothesis; (D) other hypothesis.

The origin of the winged insects is one of the most fascinating questions in the evolutionary biology of invertebrates. A current controversy is the phylogenetic relationship between the Ephemeroptera (mayfly) and Odonata (dragonfly and damselfly) that is hotly debated by taxonomists and systematists. Three main hypotheses have been proposed to explain the phylogenetic position of Ephemeroptera and Odonata based on morphological characteristics. The first hypothesis, termed the Palaeoptera hypothesis, suggests that Palaeoptera (=Ephemeroptera + Odonata) is the sister group of Neoptera based on characteristics including their inability to fold wings back over the abdomen, and the possession of an anal brace, bristle-like antennae, and intercalary veins that are exclusive to Palaeoptera (Bechly et al., 2001; Blanke et al., 2012; Hennig, 1981; Kukalová-Peck, 1991; Staniczek, 2000). The second hypothesis, termed the Metapterygota hypothesis (=Odonata + Neoptera), suggests that Ephemeroptera is the sister group to other winged insects based on the presence of a subimago, the caudal filament, absence of basalar-sternal muscles, and locking fixation of the anterior mandibular articulation (Grimaldi & Engel, 2005; Kristensen, 1981). The third hypothesis, termed the Chiastomyaria hypothesis (= Ephemeroptera + Neoptera), suggests that Odonata is the sister group to other winged insects due to a lack of direct sperm transfer among its species (Boudreaux, 1979; Matsuda, 1970). Molecular studies have provided support for each of three hypotheses even when using the same datasets. Results from different molecular datasets (e.g., mitochondrial genes, nuclear genes, mitochondrial genomes, transcriptomes, or whole genomes) are also not in consensus (Cai et al., 2018; Giribet & Ribera, 2000; Hovmöller, Pape & Källersjö, 2002; Kjer, 2004; Li, Qin & Zhou, 2014; Lin, Chen & Huang, 2010; Meusemann et al., 2010; Misof et al., 2007; Misof et al., 2014; Ogden & Whiting, 2003; Ogden & Whiting, 2005; Regier et al., 2010; Simon et al., 2009; Simon, Schierwater & Hadrys, 2010; Simon et al., 2012; Simon & Hadrys, 2013; Song et al., 2019; Wheeler et al., 2001; Zhang, Song & Zhou, 2008a; Zhang et al., 2010). Molecular studies in support of each of these three hypotheses are shown in Fig. 1 and Table S1, along with the outgroups and methods used in each study. In addition, some molecular datasets using similar gene datasets but different analysis methods support different hypotheses (Hovmöller, Pape & Källersjö, 2002; Meusemann et al., 2010; Hovmöller, Pape & Källersjö, 2002; Meusemann et al., 2010; Ogden & Whiting, 2003; Simon et al., 2009). Support for the Metapterygota hypothesis came from the use mitochondrial (mt) genomes (Zhang et al., 2008b; Cai et al., 2018). However, Zhang et al. (2010) and Lin, Chen & Huang (2010) suggested that Odonata was a sister to other Pterygota via analysis of mt genomes. Thomas et al. (2013) reanalyzed the dataset of Lin, Chen & Huang (2010) using the BEAST method and recovered Metapterygota, consistent with the original findings of Zhang et al. (2008b). Song et al. (2019) supported the Palaeoptera hypothesis using mt genomes. Whitfield & Kjer (2008) pointed out that an “ancient rapid radiation” was likely a major contributing factor to the unresolved relationship of winged insects using molecular datasets. However, only a few representatives from Odonata and Ephemeroptera were used in each of the previous phylogenetic studies of pterygotes (Misof et al., 2014; Regier et al., 2010; Zhang et al., 2008b; Zhang et al., 2010). Most of these studies also examined relationships using different outgroup taxa even when using similar inference methods (Table S1). Rota-Stabelli & Telford (2008) have suggested that the choice of an appropriate outgroup is a fundamental prerequisite when the differences between two conflicting phylogenetic hypotheses depends on the position of the root. Hence, the question arises: if we increase taxon sampling and choose appropriate outgroups, can this give us enough information to obtain an accurate phylogeny?

The mt genome is a circular molecule of sizes ranging from approximately 15 to 20 kb and encodes 2 rRNA genes, 22 tRNA genes, 13 protein-coding genes (PCGs), and an A+T-rich control region (Boore, 1999; Cameron, 2014). Phylogenetic analyses of insect mt genomes have indicated that mt PCGs are informative and useful sources of intra-order or lower level relationships (Cameron, 2014; Cameron & Whiting, 2008; Cheng et al., 2016; Kjer et al., 2016; Ma et al., 2015; Song et al., 2016; Wang et al., 2019; Zhang et al., 2018). With the aim of reconstructing the phylogenetic relationships of primordial winged insect orders, we sequenced 28 complete or nearly complete mt genomes from Ephemeroptera (18 species), Odonata (eight species) and Plecoptera (two species). We also analyzed the mt gene arrangement characters and the phylogenetic relationships within Odonata and Ephemeroptera at the family-level.

## Material and Methods

### Ethical statement

No invertebrate specimens used in this study are protected under the provisions of the laws of People’s Republic of China on the protection of wildlife. Hence, there is no ethical problem with animal sampling. The study protocol was reviewed and approved by the Committee of Animal Research Ethics of Zhejiang Normal University.

### Samples and sequencing

Mitochondrial genomes from 28 species of mayflies, dragonflies, damselflies and stoneflies were sequenced. All samples were collected between 2005 and 2014; information on species collected are available in Table 1. All mayflies and stoneflies were collected at the larval stage, whereas the dragonflies (or damselflies) were collected at the adult stage. All samples were preserved in 100% or 85% ethanol and stored at −40 °C in the Zhang‘s lab, College of Life Science and Chemistry, Zhejiang Normal University, China. All samples were identified by JY Zhang based on morphological characteristics. DNA was extracted from either a whole individual (for smaller species) or from the legs of an individual (for larger species) using DNeasy Tissue Kits (Qiagen, Hilden, Germany). For each sample we amplified short 400–1,500 bp mt gene fragments using degenerate primers designed to match specific arthropod mt genes according to the method of Simon et al. (2006). PCR reaction conditions and procedures were as described in Zhang, Song & Zhou (2008a), Zhang et al. (2008b), Yu et al. (2016). Amplification parameters were not stringent (46−58 °C annealing temperatures). Short fragments were sequenced using the ABI 3730 system with bidirectional sequencing. Species-specific primers were then designed from these short fragments and used to amplify longer 2–3 kb segments of the mt genome from each sample. Long PCR products were sequenced directly using a primer-walking strategy initiated with specific primers with bidirectional sequencing. Raw sequence files were proofread manually and assembly of the nearly complete genome sequence was performed in SeqMan of Lasergene version 5.0 (Burland, 1999). Thirteen protein-coding genes (PCGs) and 2 rRNA (*16S rRNA* and *12S rRNA*) genes were identified by comparisons with homologous sequences of known insect mt genes. Most tRNA genes were identified by their cloverleaf secondary structure using tRNAscan-SE 1.21 (Lowe & Eddy, 1997). If selected tRNA genes could not be determined by tRNAscan-SE, they were identified by comparison with the homologous insect tRNA genes.

**Table 1:** Information on specimen sources of the samples used in this study.

Sample Number	Species	Order	Family	Location of collection
TXF2014010	*Epeorus* sp.	Ephemeroptera	Heptageniidae	Jinzhai, Anhui
FSJBF2010002	*Heptagenia* sp.	Ephemeroptera	Heptageniidae	Jinhua, Zhejiang
FSJF2014001	*Iron* sp.	Ephemeroptera	Heptageniidae	Jinhua, Zhejiang
AJXF2014001	*Cinygmina obliquistrita*	Ephemeroptera	Heptageniidae	Jinzhai, Anhui
SXF2011004	Ephemerellidae sp.	Ephemeroptera	Ephemerellidae	Sangzhi, Hubei
AYWF2014005	*Drunella* sp.	Ephemeroptera	Ephemerellidae	Yuexi, Anhui
JSTF2010004	*Uracanthella* sp.	Ephemeroptera	Ephemerellidae	Shangrao, Jiangxi
JXF2014002	*Serratella* sp.	Ephemeroptera	Ephemerellidae	Jinzhai, Anhui
AHWF2014070	*Rhoenanthus* sp.	Ephemeroptera	Potamanthidae	Huoshan, Anhui
HGHF2011011	*Potamanthus kwangsiensis*	Ephemeroptera	Potamanthidae	Sangzhi, Hubei
AJDF2014110	*Isonychia ignota*	Ephemeroptera	Isonychiidae	Jinzhai, Anhui
JSZF2010111	*Caenis* sp.	Ephemeroptera	Caenidae	Shangrao, Jiangxi
JAXXCF2010090	*Leptophlebia* sp.	Ephemeroptera	Leptophlebiidae	Jinhua, Zhejiang
JSMF2010046	*Ephemera shengmi*	Ephemeroptera	Ephemeridae	Shangrao, Jiangxi
HBF2010057	*Ephemera* sp.	Ephemeroptera	Ephemeridae	Jinhua, Zhejiang
AHYNF2014036	*Vietnamella* sp.	Ephemeroptera	Austremerellidae	Jinzhai, Anhui
HHF2011069	*Ephoron yunnanensis*	Ephemeroptera	Polymitarcyidae	Banna, Yunnan
NSF2005069	*Siphluriscus chinensis*	Ephemeroptera	Siphluriscidae	Leishan, Guizhou
QTSC2010051	*Mnais* sp.	Odonata	Calopterygidae	Lanxi, Zhejiang
SC2006001	*Platycnemis phyllopoda*	Odonata	Platycnemididae	Nanjing, Jiangsu
HC2006051	*Ceriagrion nipponicum*	Odonata	Coenagrionidae	Nanjing, Jiangsu
CT2006032	*Anisogomphus maacki*	Odonata	Gomphidae;	Nanjing, Jiangsu
YDQ2012020	*Pseudothemis zonata*	Odonata	Libellulidae	Jinhua, Zhejiang
ZFQ2012034	*Acisoma panorpoides*	Odonata	Libellulidae	Zhoushan, Zhejiang
AJHQ2014065	*Pantala flavescens*	Odonata	Libellulidae	Jinzhai, Anhui
DQ2010009	*Neallogaster pekinensis*	Odonata	Cordulegastridae	Jinhua, Zhejiang
WQSJK2010108	Perlidae sp.	Plecoptera	Perlidae	Jinhua, Zhejiang
FSJCJ20100107	*Nemoura* sp.	Plecoptera	Nemouridae	Jinhua, Zhejiang

### Taxa and alignment

In previous phylogenetic analyses some insect species have been reported to suffer from long-branch attraction and to have unstable phylogenetic positions (Nardi et al., 2003). Although many mt genomes from all orders of Insecta can now be acquired from GenBank data, we did not include data from those orders whose species showed long-branch attraction in previous phylogenetic analyses or high gene rearrangements or high base compositional biases which can cause erroneous results (Bergsten, 2005; Castro & Dowton, 2005; Ma et al., 2014; Wan et al., 2012). To reduce the computational analysis time we choose 1–5 species of the Diptera, Dermaptera, Lepidoptera, Orthoptera, Mantodea, Phasmida, Blattodea, Isoptera (=Blattodea: Termitoidae), Megaloptera, Neuroptera, Mecoptera, Grylloblattodea, Mantophasmatodea and Coleoptera as the ingroup. The outgroups selected were the four available bristletail sequences (Archaeognatha) ((*Nesomachilis australica* (Cameron et al., 2004), *Pedetontus silvestrii* (Zhang, Song & Zhou, 2008a), *Petrobius brevistylis* (Podsiadlowski, 2006), *Trigoniophthalmus alternatus* (Carapelli et al., 2007)) and three silverfishes (Thysanura) (*Atelura formicaria* (Comandi et al., 2009)), *Tricholepidion gertschi* (Nardi et al., 2003), *Thermobia domestica* (Cook, Yue & Akam, 2005)). We combined the 28 newly-sequenced mt genomes with 85 species from 19 orders of Insecta obtained from GenBank (Table 2). The ATP8 gene was not used in the subsequent analyses due to its shortness (about 50 amino acid residues) and its poor conservation (Zhang et al., 2008b). We aligned amino acid sequences of each mt protein-encoding gene separately. The dataset was composed of 113 taxa, including 4 bristletails, 3 springtails, 13 odonatans, 16 mayflies, 10 stoneflies and 39 neopteran species, all retrieved from GenBank (Table 2). We used two trivially different alignment methods (Cluster W and Muscle in Mega 7.0 (Kumar, Stecher & Tamura, 2016)). To reduce bias we deleted the nonconserved regions in each of gene alignment using GBlocks 0.91b (Castresana, 2000) with block parameters set as default. The two alignments and block identification procedures ensured that the conserved regions were reliably homologous. Each of the conserved alignments were concatenated for both nucleotide and amino acid translated datasets, a final alignment 7011 nucleotides and 2337 amino acids residues. A saturation analysis of the concatenated DNA was performed for first, second and third codon positions using DAMBE4.2.13 (Xia & Xie, 2001). Third codon positions were saturated so they were excluded from the final alignment and the final alignment 4674 including first and second codon positions of 113 sequences were obtained (named 113 dataset).

**Table 2:** GenBank numbers of the 113 taxa of insects in this study.

Order	Species	Accession no.	Reference
Archaeognatha	*Pedetontus silvestrii*	EU621793	Zhang, Song & Zhou (2008a)
	*Petrobius brevistylis*	AY956355	Podsiadlowski (2006)
	*Petrobiellus puerensis*	KJ754503	Ma et al. (2015)
	*Trigoniophthalmus alternatus*	EU016193	Carapelli et al. (2007)
	*Nesomachilis australica*	AY793551	Cameron et al. (2004)
Zygentoma	*Tricholepidion gertschi*	AY191994	Nardi et al. (2003)
	*Atelura formicaria*	EU084035	Comandi et al. (2009)
	*Thermobia domestica*	AY639935	Cook, Yue & Akam (2005)
Odonata	*Ischnura pumilio*	KC878732	Lorenzo-Carballa (2014)
	*Platycnemis phyllopoda*	MF352167	this study
	*Pseudolestes mirabilis*	FJ606784	unpublished
	*Ceriagrion nipponicum*	MF352157	this study
	*Euphaea formosa*	HM126547	Lin, Chen & Huang (2010)
	*Euphaea ornate*	KF718295	unpublished
	*Euphaea yayeyamana*	KF718293	unpublished
	*Euphaea decorata*	KF718294	unpublished
	*Mnais* sp.	MF352166	this study
	*Vestalis melania*	JX050224	unpublished
	*Atrocalopteryx atrata*	KP233805	unpublished
	*Ictinogomphus* sp.	KM244673	Comandi et al. (2009)
	*Davidius lunatus*	EU591677	Lee et al. (2009)
	*Anisogomohus maacki*	MF352151	this study
	*Pseudothemis zonata*	MF352170	this study
	*Pantala flavescens*	MF352148	this study
	*Acisoma panorpoides*	MF352171	this study
	*Orthetrum triangulare*	AB126005	Yamauchi, Miya & Nishida (2004)
	*Cordulia aenea*	JX963627	unpublished
	*Hydrobasileus croceus*	KM244659	Tang et al. (2014)
	*Neallogaster pekinensis*	MF352152	this study
Ephemeroptera	*Siphluriscus chinensis*	HQ875717	Li, Qin & Zhou (2014)
	*Siphluriscus chinensis*	MF352165	this study
	*Ameletus* sp.	KM244682	Tang et al. (2014)
	*Siphlonurus* sp.	KM244684	Tang et al. (2014)
	*Parafronurus youi*	EU349015	Zhang, Song & Zhou (2008a) and Zhang et al. (2008b)
	*Isonychia ignota*	HM143892	unpublished
	*Epeorus* sp.MT-2014	KM244708	Tang et al. (2014)
	*Epeorus* sp.JZ-2014	KJ493406	this study
	*Isonychia ignota*	MF352147	this study
	*Paegniodes cupulatus*	HM004123	unpublished
	*Iron* sp.	MF352155	this study
	*Heptagenia* sp.	MF352153	this study
	*Cinygmina obliquistrita*	MF352149	this study
	*Ephoron yunnanensis*	MF352159	this study
	*Habrophlebiodes zijinensis*	GU936203	unpublished
	*Ephemera orientalis*	EU591678	Lee et al. (2009)
	*Ephemera* sp.	MF352156	this study
	*Ephemera shengmi*	MF352161	this study
	*Leptophlebia* sp.	MF352160	this study
	*Siphlonurus immanis*	FJ606783	unpublished
	*Potamanthus* sp.MT-2014	KM244674	Tang et al. (2014)
	*Rhoenanthus* sp.	MF352145	this study
	*Potamanthus kwangsiensis*	MF352158	this study
	*Caenis* sp. QY-2009	GQ502451	unpublished
	*Caenis* sp.	MF352163	this study
	Teloganodidae sp.MT-2014	KM244703	Tang et al. (2014)
	*Ephemerella* sp.MT-2014	KM244691	Tang et al. (2014)
	*Drunella* sp.	MF352150	this study
	*Vietnamella dabieshanensis*	HM067837	unpublished
	*Vietnamella* sp.	KM244655	Tang et al. (2014)
	*Vietnamella* sp.JZ-2017	MF352146	this study
	Ephemerellidae sp.	MF352168	this study
	*Serratella* sp.	MF352164	this study
	*Uracanthella* sp.	MF352162	this study
Plecoptera	*Acroneuria hainana*	KM199685	Huang et al. (2015)
	*Togoperla* sp.	KM409708	Wang, Liu & Yang (2014)
	*Kamimuria wangi*	KC894944	unpublished
	Perlidae sp.	MF352169	this study
	*Dinocras cephalotes*	KF484757	Elbrecht et al. (2015)
	*Apteroperla tikumana*	KR604721	Unpublished
	*Nemoura* sp.	MF352154	this study
	*Styloperla* sp.	KR088971	Chen, Wu & Du (2016)
	*Pteronarcella badia*	KU182360	Sproul et al. (2015)
	*Pteronarcys princeps*	AY687866	Stewart & Beckenbach (2006)
	*Mesocapnia arizonensis*	KP642637	unpublished
	*Cryptoperla* sp.WX-2013	KC952026	Wu et al. (2014)
Phasmatodea	*Timema californicum*	DQ241799	Cameron, Barker & Whiting (2006)
Mantophasmatodea	*Sclerophasma paresisensis*	DQ241798	Cameron, Barker & Whiting (2006)
Grylloblattodea	*Grylloblatta sculleni*	DQ241796	Cameron, Barker & Whiting (2006)
Mantodea	*Tamolanica tamolana*	DQ241797	Cameron, Barker & Whiting (2006)
	*Leptomantella albella*	KJ463364	Wang, Wang & Yang (2016)
Blattodea	*Blattella germanica*	EU854321	Xiao et al. (2012)
	*Periplaneta fuliginosa*	AB126004	Yamauchi, Miya & Nishida (2004)
	*Eupolyphaga sinensis*	FJ830540	Zhang et al. (2010)
Termitoidae	*Coptotermes formosanus*	KU925203	Bourguignon et al. (2016)
	*Reticulitermes flavipes*	EF206317	Cameron & Whiting (2007)
Diptera	*Drosophila melanogaster*	DMU37541	Clary et al. (1982)
	*Chrysomya putoria*	AF352790	Junqueira et al. (2004)
	*Simosyrphus grandicornis*	DQ866050	Cameron et al. (2007)
Mecoptera	*Neopanorpa pulchra*	FJ169955	unpublished
Raphidioptera	*Mongoloraphidia harmandi*	FJ859902	Cameron et al. (2009)
Coleoptera	*Pyrophorus divergens*	EF398270	Arnoldi et al. (2007)
	*Chaetosoma scaritides*	EU877951	Sheffield et al. (2008)
Neuroptera	*Ditaxis biseriata*	FJ859906	Cameron et al. (2009)
	*Polystoechotes punctatus*	FJ171325	Beckenbach & Stewart (2009)
Megaloptera	*Sialis hamata*	FJ859905	Cameron et al. (2009)
	*Corydalus cornutus*	FJ171323	Beckenbach & Stewart (2009)
	*Protohermes concolorus*	EU526394	Hua et al. (2009)
Orthoptera	*Gastrimargus marmoratus*	EU513373	Ma et al. (2009)
	*Teleogryllus oceanicus*	KT824636	unpublished
	*Teleogryllus emma*	EU557269	unpublished
	*Velarifictorus hemelytrus*	KU562918	Yang, Ren & Huang (2016)
	*Loxoblemmus equestris*	KU562919	Yang, Ren & Huang (2016)
	*Locusta migratoria*	X80245	Flook, Rowell & Gellissen (1995)
Trichoptera	*Eubasilissa regina*	KF756943	Wang, Liu & Yang (2014)
	*Limnephilus decipiens*	AB971912	unpublished
Lepidoptera	*Saturnia boisduvalii*	EF622227	Hong et al. (2008)
	*Manduca sexta*	EU286785	Cameron & Whiting (2008)
	*Bombyx mandarina*	GU966631	Li et al. (2010)
Hemiptera	*Triatoma dimidiata*	AF301594	Dotson & Beard (2001)
	*Homalodisca coagulata*	AY875213	unpublished
	*Nezara viridula*	EF208087	Hua et al. (2008)
Dermaptera	*Euborellia arcanum*	KX673196	Song et al. (2016)
	*Labidura japonica*	KX673201	Song et al. (2016)
	*Challia fletcheri*	JN651407	Wan et al. (2012)

To compare the effect of outgroup choice, we added and analyzed two additional combinations of outgroup species: 114 dataset selecting five bristletails (*Nesomachilis australica*, *Pedetontus silvestrii*, *Petrobius brevistylis*, *Trigoniophthalmus alternatus*, and *Petrobiellus puerensis*) as outgroups according to the Metapterygota hypothesis supported by Cai et al. (2018); 119 dataset selecting five diplurans (*Campodea lubbocki*, *C. fragilis*, *Japyx solifugus*, *Lepidocampa weberi*, and *Occasjapyx japonicus*) as outgroups according to the Palaeoptera hypothesis supported by Song et al. (2019). The conserved region and saturation analyses are similar to the method used for the 113 dataset. The dataset with five bristletails as outgroups and the dataset with five diplurans as outgroups were named the 114 dataset and 119 dataset, respectively.

### Phylogenetic analyses

PartitionFinder ver. 1.1.1 (Lanfear et al., 2012) was used to select partitions using the Bayesian Information Criterion (BIC). The 24 parts of three nucleotide datasets were divided into 9 partitions. The best model of three datasets was identified as a general time-reversible model (GTR+I+G) in all partitions excluding a TVM+I+ model in the second position of the COIII gene partition, whereas the second best model of this was the GTR+I+G using ModelGenerator v0.85 (Keane et al., 2006). To deduce differences in phylogenetic analyses we used a GTR+I+G model with first and second positions of 12 mt genes in three nucleotide datasets. We used MrBayes v. 3.2 (Ronquist et al., 2012) to estimate phylogenies by Bayesian inference (BI) for each dataset. Two independent runs of four incrementally heated Markov chain Monte Carlo (MCMC) chains (one cold chain and three hot chains) were simultaneously run for ten million generations depending on the datasets, with sampling conducted every 100 generations. The convergence of MCMC, which was monitored by the average standard deviation of split frequencies, reached below 0.01 within ten million generations depending on the dataset, and the initial 25% of the sampled trees were discarded as burn-in. Bayesian posterior probabilities were calculated from the remaining set of trees. Maximum likelihood (ML) analysis was conducted using PhyML (Guindon et al., 2005), specifying four substitution rate and the corresponding BI tree as the start tree. The confidence values of the ML tree were evaluated via a bootstrap test with 100 iterations.

## Results

### Genome organization of mtDNA

**Figure 2:**
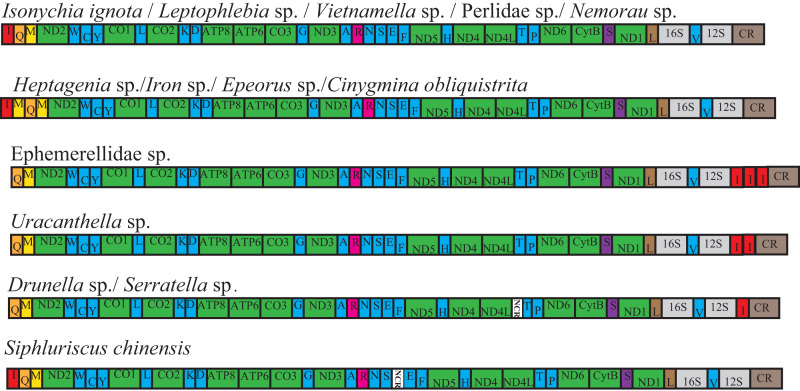
Fourteen complete mitochondrial genome maps of the Ephemeroptera species used in this study. The tRNAs are labeled according to the single-letter amino acid codes. The gene name above the median indicates the direction of transcription is from left to right, whereas the gene name below the median indicates right to left.

Of the 28 complete or nearly mt genomes of Ephemeroptera, Odonata and Plecoptera, four species of Ephemeroptera were found to contain a gene rearrangement, and four species of Ephemeroptera contained a tRNA gene duplication (Fig. 2). Four species from Heptageniidae (*Cinygmina obliquistrita*, *Epeorus* sp., *Heptagenia* sp. and *Iron* sp.) had an extra *tRNA-Met* gene located between *tRNA-Ile* and *tRNA-Gln*. Four species from Ephemerellidae (*Drunella* sp., *Serratella* sp., *Uracanthella* sp. and Ephemerellidae sp.) had a *tRNA-Ile* gene translocated and inversed to a position between 12S RNA and the control region. One copy of *tRNA-Ile* was present in *Drunella* sp. and *Serratella* sp., whereas two copies of this gene were present in *Uracanthella* sp. and three copies in Ephemerellidae sp.

### Phylogenetic analyses of three datasets

**Figure 3:**
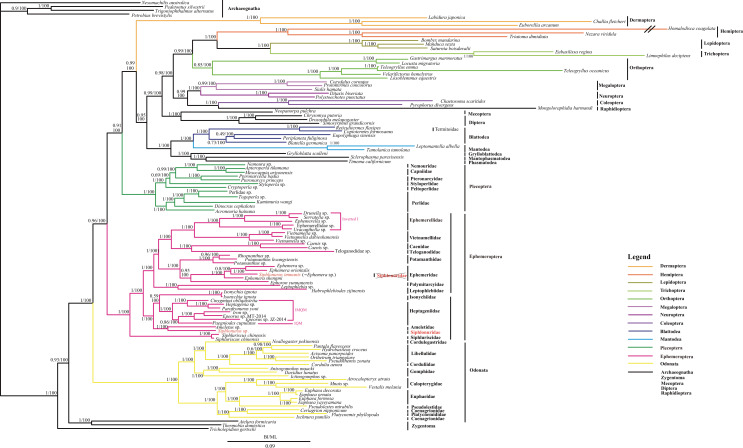
The BI and ML phylogenetic relationships of Ephemeroptera, Odonata and Neoptera as assessed from 12 protein-coding genes using nucleotide data 113FY7. Phylogenetic analyses using nucleotide data were carried out for the 113 insect species based on all 12 protein-coding genes from their respective mt genomes. Four bristletails (*Pedetontus silvestri*, *Petrobius brevistylis*, *Trigoniophthalmus alternatus*, *Nesomachilis australica*) and three silverfishes (*Atelura formicaria*, *Tricholepidion gertschi*, *Thermobia domestic*) were used as outgroups. Numbers above the nodes are the posterior probabilities of BI and the bootstrap values of ML.

We employed BI and ML analyses to construct phylogenetic trees from three datasets with different outgroups (Fig. 3 and Figs. S1–S4). We found that different outgroups can affect the relationships between Ephemeroptera, Odonata and Neoptera. The Chiastomyaria hypothesis was strongly supported in BI analyses of three outgroup datasets and ML analysis of the 113 dataset (Fig. 3 and Figs. S1–S2), whereas different results in ML analyses of the 114 and 119 datasets were found (Figs. S3–S4). ML analyses in 114 and 119 datasets failed to support the Chiastomyaria hypothesis. No support for either the Palaeoptera or Metapterygota hypotheses was found in the BI and ML analyses in this study.

Comparing the phylogenetic trees derived from the three datasets with different outgroups, we found that the 113 dataset using 4 bristletails and 3 silverfishes as the outgroups is the stable phylogeny with high posterior probability and bootstrap value. In the 113 dataset, the monophyly of Odonata, Ephemeroptera and Neoptera was strongly supported in both BI and ML analyses (Fig. 3). Odonata as the sister of the remaining Pterygota had high posterior probability (1.00) and bootstrap value (100). Ephemeroptera as the sister of Neoptera also showed high support (BI/ML: 1.00/100) and Plecoptera was sister to the remaining other Neoptera with high support (0.92/100).

Within Odonata, the monophyly of the suborders Zygoptera and Anisoptera was well supported. At the family level, the monophyly of Gomphidae, Calopterygidae, Euphaeidae, and Libellulidae was well supported but the monophyly of Coenagrionidae was not supported since Platycnemididae clustered within Coenagrionidae.

Within Ephemeroptera, *Siphluriscus chinensis* (Siphluriscidae) was sister to the other mayflies, which were divided into two clades. All nodes had high posterior probabilities excluding (Heptageniidae + Isonychiidae) which was 0.59. The monophyly of all families excluding Siphlonuridae was well supported. The monophyly of *Siphlonurus* (Siphlonuridae) was not supported because *Siphlonurus immanis* (Siphlonuridae) (FJ606783) clustered within *Ephemera* (Ephemeridae) whereas *Siphlonurus* sp. (Siphlonuridae) was a sister clade to *Ameletus* sp. (Ameletidae).

Within Neoptera, the monophyly of Plecoptera, Mantodea, Blattodea (including Isoptera), Diptera, Coleoptera, Neuroptera, Megaloptera, Orthoptera, Trichoptera, Lepidoptera, Hemiptera, and Dermaptera were all supported. Polyneoptera was not monophyletic as Plecoptera was sister to the remaining Neoptera and in turn Dermaptera was sister to Neoptera except Plecoptera. Orthoptera failed to cluster with the other orders typically assigned to Polyneoptera and was sister to Amphiesmenoptera (Lepidoptera + Trichoptera). The relationship of (Plecoptera + (Dermaptera + other Neoptera)) was strongly supported. The other two datasets generated from the different outgroups in BI analyses (Figs. S1–S2) further supported the relationship of (Odonata + (Ephemeroptera + (Plecoptera + other orders of Neoptera)). All the topology of inter-order and intra-order in BI analyses of 114 and 119 datasets was coincident with the 113 dataset except posterior probabilities and branch length so we just showed the topology of intra-order level.

## Discussion

### The mtDNA rearrangement and rearrangement mechanisms

**Figure 4:**
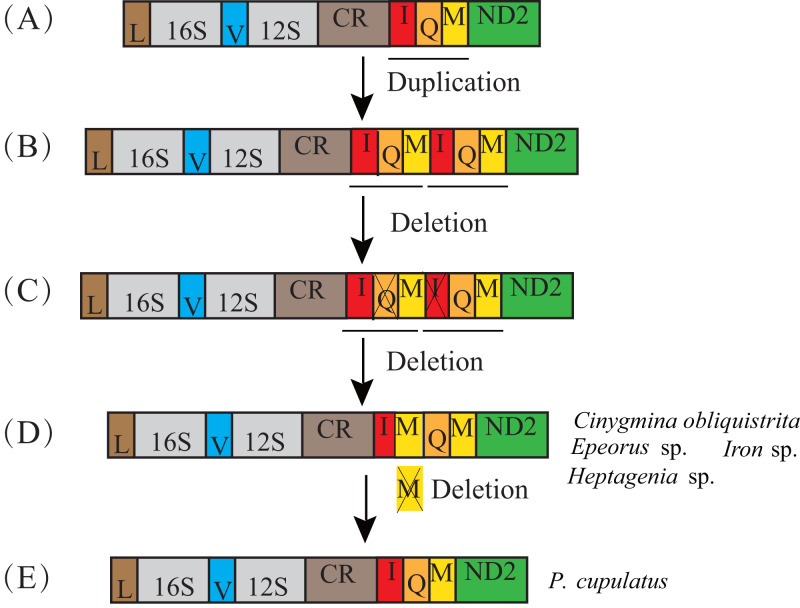
Proposed mechanism for the formation of an extra *trnM* gene in Heptageniidae under a tandem duplication and random loss model. (A) Typical insect gene order. (B) Tandem duplication in the area of *trnI-trnQ-trnM*. (C) Subsequent deletions of *trnQ* between *trnI* and *trnM*, and *trnI* between *trnM* and *trnQ*. (D) *trnI*-*trnM-trnQ-trnM cluster* formed in *Cinygmina obliquistrita*, *Epeorus* sp., *Iron* sp. and *Heptagenia* sp. (E) Subsequent deletion of *trnM* between *trnI* and *trnQ* and *trnI*-*trnQ-trnM* formed again. The gene name above the median indicates the direction of transcription is from left to right, whereas the gene name below the median indicates right to left.

In the previously published mt genomes of Ephemeroptera, an extra *tRNA-Met* gene was found in the heptageniids, *Parafronurus youi* (Zhang et al., 2008b), *Epeorus* sp. (Tang et al., 2014), and *Epeorus herklotsi* (Gao et al., 2018), but was not found in *Paegniodes cupulatus* (Zhou et al., 2016). In the current study, *Cinygmina obliquistrita*, *Epeorus* sp., *Heptagenia* sp. and *Iron* sp. (Heptageniidae) all had the extra *tRNA-Met*. Based on the all species excluding *P. cupulatus* possessing an extra *tRNA-Met* (Fig. 3), we deduce that the common ancestor of the Heptageniidae had an extra *tRNA-Met* gene, which can be explained as well as *P. youi* through the tandem duplication-random loss (TDRL) model (Zhang et al., 2008b). However, the extra *tRNA-Met* gene in *P. cupulatus* was apparently deleted in an independent random loss thereby restoring the *IQM* tRNA gene cluster (Fig. 4).

**Figure 5:**
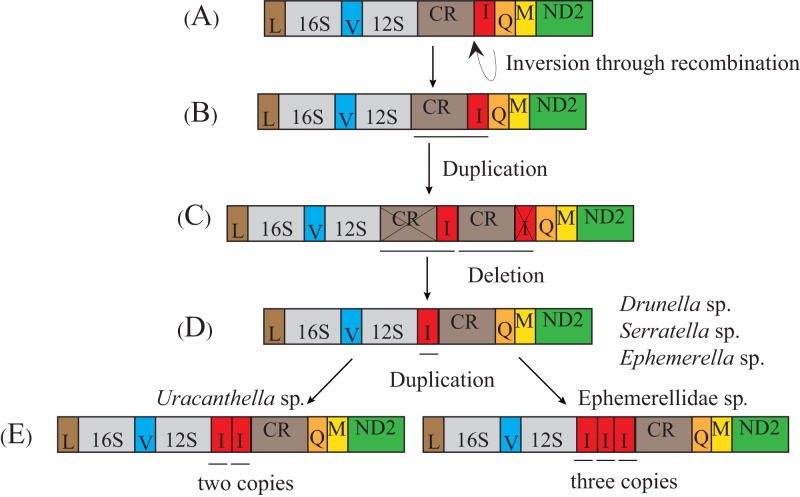
Proposed mechanism of gene rearrangements of the *trnI* gene in Ephemerellidae under a model of tandem duplication of gene regions and recombination. (A) Typical insect gene order. (B) Tandem duplication in the area from the control region (CR) to *trnI*. (C) Subsequent deletions of CR and *trnI* between CR and *trnQ* gene. (D) *trnI* gene inverted through recombination. (E) Tandem duplication of inverted *trnI*. The gene name above the median indicates the direction of transcription is from left to right, whereas the gene name below the median indicates right to left.

The transposition and inversion of *tRNA-Ile* genes in the mt genome of the Ephemerellidae can also be explained by the recombination and tandem duplication-random loss (TDRL) model (Fig. 5). Firstly, the *tRNA-Ile* inversion in Ephemerellidae may have been caused by a recombination in the original location, involving the breakage and rejoining of participating *tRNA-Ile* (Lunt & Hyman, 1997). Secondly, a *tRNA-Ile* inversion-CR arrangement probably occurred by duplication of the CR-*tRNA-Ile* inversion regions, resulting in a CR-*tRNA-Ile* inversion-CR-*tRNA-Ile* inversion arrangement, and then deleted by a subsequent random loss of the first copy CR and the second copy *tRNA-Ile* gene. At this point, it can be explained by a typical TDRL model (Moritz, Dowling & Brown, 1987). One copy of the *tRNA-Ile* gene inversion was formed in the ancestor of *Drunella* sp. and *Serratella* sp. as well as *Ephemerella* sp. (Ephemerellidae) (Tang et al., 2014), *Cincticostella fusca* (Ephemerellidae) (Li et al., 2020) and species of *Serratella* (Ephemerellidae) (Xu et al., 2020b), but three copies of the *tRNA-Ile* inversion in Ephemerellidae sp. as well as species of *Torleya* (Ephemerellidae) (Xu et al., 2020a; Xu et al., 2020b) and two copies of the *tRNA-Ile* in *Uracanthella* sp. were formed through different random duplications of the *tRNA-Ile* gene inversion.

### Phylogenetic analyses and the Chiastomyaria hypothesis

Each of the three hypotheses, Palaeoptera, Chiastomyaria or Metapterygota, have been supported in different studies using molecular datasets and various analysis methods (see Table S1). In our results, the use of increased numbers of mayfly, dragonfly, damselfly, and stonefly mt genomes sheds light on the phylogenetic origins of winged insects. Although outgroup choice may affect the phylogenetic relationships of Ephemeroptera and Odonata, we recovered support for the Chiastomyaria hypothesis with the choice of different outgroups. The hypothesis of Odonata as the origin of winged insects was also supported by Kjer (2004), Wan et al. (2012), Misof et al. (2014), Simon et al. (2012), Li, Qin & Zhou (2014). But some researchers supported the Metapterygota hypothesis using the mitochondrial genomes method (Tang et al., 2014; Cai et al., 2018; Zhang, Song & Zhou, 2008a; Zhang et al., 2008b).

Suitable outgroups are very important to determining phylogenetic relationships in the rapid evolution of insects. Hence, we must carefully choose the appropriate outgroups in order to discuss the relationships of Ephemeroptera and Odonata. According to the three outgroups tested in the current study, we propose that four bristletails and three silverfishes together is an ideal outgroup to use to evaluate the phylogenetic relationship of Ephemeroptera and Odonata. When we chose four bristletails and three silverfishes as the outgroups (113 dataset), the Chiastomyaria hypothesis was supported in ML and BI analyses (Fig. 3). When we chose five bristletails and three silverfishes as the outgroups (114 dataset), the Chiastomyaria hypothesis was only supported in BI analysis (Fig. S1), whereas the phylogenetic relationship of Ephemeroptera and Odonata failed to recover and formed an different relationship in PhyML analysis (Fig. S3). However, Cai et al. (2018) supported the Metapterygota hypothesis using the same outgroups (five bristletails and three silverfishes). When we used five diplurans as outgroups we also found strong support for the Chiastomyaria hypothesis in BI analysis (Fig. S2), whereas the phylogenetic relationship of Ephemeroptera and Odonata failed to recover in PhyML analysis (Fig. S4). Song et al. (2019) supported the Palaeoptera hypothesis using two species of Collembola and five species of Diplura as outgroups. We compared the outgroups used in published papers (Table S1) and found that few studies use the same outgroups. Thomas et al. (2013) thought that using few suitable outgroups or only distant outgroups may cause systematic error even in large datasets. Despite testing three outgroup combinations and finding support for Chiastomyaria hypothesis, the possibility still exists of bias in outgroup choice.

### The phylogenetic family-level relationships in Odonata and Ephemeroptera

The phylogenetic relationships revealed among Odonata families in the current study are similar to the results of Yong et al. (2016) and Dijkstra et al. (2014). We demonstrated that the mt genome was a suitable marker to discuss the family-level phylogenetic relationships of Odonata, as also reported by Yong et al. (2016). However, the monophyly of Coenagrionidae was not supported in our study.

In the phylogenetic relationships at the family level, *Siphluriscus chinensis* (Siphluriscidae) was supported as sister to the rest of the Ephemeroptera as in the previous results of Ogden et al. (2009), Li, Qin & Zhou (2014), and Zhou & Peters (2003). Isonychiidae was a sister group to Heptageniidae, making the Suborder Setisura (=Heptagenioidea) as supported by McCafferty (1991), whereas Ogden & Whiting (2005), Ogden et al. (2009), Gao et al. (2018), Ye et al. (2018), Cao et al. (2020), and Xu et al. (2020a) supported Isonychiidae as sister to most Ephemeroptera (excluding Baetidae and Siphluriscidae).

The monophyly of Ephemerellidae with different copies of the *tRNA-Ile* inversion is well supported in this study. Our study identifies *Ephemerella* sp. as the sister group to the clade of *Drunella* sp. and *Serratella* sp. with one copy of the *tRNA-Ile* inversion. Ephemerellidae sp. with three copies of the *tRNA-Ile* inversion is the sister group to *Uracanthella* sp. with two copies. Ogden et al. (2009) found that *Ephemerella* and *Serratella* were not supported as monophyletic. In the present study we found a *tRNA-Ile* translocation and inversion in Ephemerellidae and a different copy of the *tRNA-Ile* inversion in *Ephemerella* and *Serratella*. This gives us more evidence to identify the monophyly of *Ephemerella* and *Serratella* in future studies. However, the mt genomes of Ephemeroptera are suitable markers to discuss the family-level phylogenetic relationship of Ephemeroptera.

### The phylogenetic relationship of Plecoptera and Dermaptera

In previous studies of Neoptera, one Plecoptera (*Pteronarcys princeps*) and one Dermaptera (*Challia fletcheri*) species were included (Li, Qin & Zhou, 2014). Results indicated that *P. princeps* was sister to Orthoptera whereas *Challia fletcheri* with a very long-branch was sister to either the rest of Polyneoptera or Ephemeroptera. When only one species of Plecoptera (*P. princeps*) was included, but Dermaptera (*C. fletcheri*) was excluded (Lin, Chen & Huang, 2010; Zhang et al., 2010), Plecoptera was sister to Ephemeroptera. However, when we increased the sampling of Plecoptera and Dermaptera, we found that Plecoptera was sister to the remaining Neoptera, and Dermaptera was sister to Neoptera excluding Plecoptera. The position of Plecoptera as sister to the rest of Neoptera and not within Polyneoptera was very interesting. Although Beutel & Gorb (2006) proposed Plecoptera to be in this position, Cai et al. (2018) and Song et al. (2019) supported the position of Plecoptera as sister to the rest of Neoptera in the phylogenetic trees of BI and ML analyses. Some researchers also supported Plecoptera as likely occupying a position near the root of the Neoptera or Polyneoptera, and the aquatic life history stage to be an ancestral feature of winged insects (Marden & Kramer, 1994; Marden, 2013; Zwick, 2009). Most other studies placed Plecoptera within Polyneoptera, often as sister to Dermaptera (e.g., Kjer, 2004; Misof et al., 2007; Simon et al., 2012; Von Reumont et al., 2009). However, Matsumura et al. (2015) supported Plecoptera as the sister group to Zoraptera. Song et al. (2016) reported that Plecoptera was sister to Dermaptera but their ingroup data included only Polyneoptera (no Holometabola and Paraneoptera). Wipfler et al. (2019) found Plecoptera was sister to Polyneoptera excluding Zoraptera and Demaptera and suggested the ancestor of winged insects did not evolve in an aquatic environment. In our study, we recovered the relationship Odonata + (Ephemeroptera + (Plecoptera + (Dermaptera + other Neoptera))) with high posterior probabilities and bootstrap values (Fig. 3). So, our results suggest that the common ancestors of the winged insects and of Neoptera were aquatic, supporting the idea that wings did evolve in an aquatic environment.

The monophyly of Polyneoptera was not supported in this study because of the placement of Plecoptera, Dermaptera and Orthoptera. Although recent studies have supported the monophyly of Polyneoptera using mt genomes or transcriptome data (Song et al., 2016; Wipfler & Pass, 2014; Wipfler et al., 2019), we found most of studies included just the Polyneopteran orders. It is suggested that such analyses should always add more species representing all insect orders, not only Polyneoptera but also including Holometabola in order to discuss the monophyly of Polyneoptera. Our analysis of the 113 dataset indicated that Orthoptera was sister to Amphiesmenoptera (Lepidoptera + Trichoptera) which may be caused by long-branch attraction of Hemiptera (Fig. 3). However, we failed to support the monophyly of Orthoptera in BI analysis of the 119 dataset.

In conclusion, we found that the mt genome is a suitable marker to investigate the phylogenetic relationship of the inter-order and inter-family relationships of insects but that the outgroup choice is very important for deriving phylogenetic relationships among winged insects. We highly recommend that we should choose suitable species from Archaeognatha and Zygentoma together as the outgroups in future research and discussions of the phylogenies of Ephemeroptera and Odonata.

##  Supplemental Information

10.7717/peerj.11402/supp-1 Supplemental Information 1 The BI phylogenetic relationships of Ephemeroptera, Odonata and Neoptera as assessed from 12 protein-coding genes using nucleotide dataset 114 Phylogenetic analyses using nucleotide data were carried out for the 114 insect species based on all 12 protein-coding genes from their respective mt genomes. Five bristletails (*Pedetontus silvestri*, *Petrobius brevistylis*, *Petrobiellus puerensis*, *Trigoniophthalmus alternatus*, *Nesomachilis australica*) and three silverfishes (*Atelura formicaria*, *Tricholepidion gertschi*, *Thermobia domestic*) were used as outgroups. Numbers above the nodes are the posterior probabilities of BI. Subtrees of the monophyly of an Order were collapsed whereas the relationship within the Order is the same as Fig. 3.

10.7717/peerj.11402/supp-2 Supplemental Information 2 The BI phylogenetic relationships of Ephemeroptera, Odonata and Neoptera as assessed from 12 protein-coding genes using nucleotide dataset 119 Phylogenetic analyses using nucleotide data were carried out for the 119 insect species based on all 12 protein-coding genes from their respective mt genomes. Five diplurans (*Campodea lubbocki*, *C. fragilis*, *Japyx solifugus*, *Lepidocampa weberi*, and *Occasjapyx japonicus*) were used as outgroups. Numbers above the nodes are the posterior probabilities of BI. Subtrees of the monophyly of the Order collapsed whereas the relationship within Order is the same as Fig. 3.

10.7717/peerj.11402/supp-3 Supplemental Information 3 The ML phylogenetic relationships of Ephemeroptera, Odonata and Neoptera as assessed from 12 protein-coding genes using nucleotide dataset 114 Phylogenetic analyses using nucleotide data were carried out for the 114 insect species based on all 12 protein-coding genes from their respective mt genomes. Five bristletails (*Pedetontus silvestri*, *Petrobius brevistylis*, *Petrobiellus puerensis*, *Trigoniophthalmus alternatus*, *Nesomachilis australica*) and three silverfishes (*Atelura formicaria*, *Tricholepidion gertschi*, *Thermobia domestic*) were used as outgroups. Numbers above the nodes are the bootstrap values of ML. Subtrees of the monophyly of the Order collapsed whereas the relationship within the Order is the same as Fig. 3.

10.7717/peerj.11402/supp-4 Supplemental Information 4 The ML phylogenetic relationships of Ephemeroptera, Odonata and Neoptera as assessed from 12 protein-coding genes using nucleotide dataset 119 Phylogenetic analyses using nucleotide data were carried out for the 119 insect species based on all 12 protein-coding genes from their respective mt genomes. Five diplurans (*Campodea lubbocki*, *C. fragilis*, *Japyx solifugus*, *Lepidocampa weberi*, and *Occasjapyx japonicus*) were used as outgroups. Numbers above the nodes are the bootstrap values of ML. Subtrees of the monophyly of the Order collapsed whereas the relationship within Order is the same as Fig. 3.

10.7717/peerj.11402/supp-5 Supplemental Information 5 References used to support the three hypotheses of Paleoptera, Metapterygota and Chiastomyaria

10.7717/peerj.11402/supp-6 Supplemental Information 6 28 mitochondrial genomes information

10.7717/peerj.11402/supp-7 Supplemental Information 7 The information of 114 alignment using the position 1, 2 of codon

10.7717/peerj.11402/supp-8 Supplemental Information 8 The information of 113 alignment using the position 1, 2, 3 of codon

10.7717/peerj.11402/supp-9 Supplemental Information 9 The information of 119 alignment using the position 1, 2 of codon

10.7717/peerj.11402/supp-10 Supplemental Information 10 The information of 113 alignment using the position 1, 2 of codon

## Additional Information and Declarations

### Data Availability

The following information was supplied regarding data availability: The mitochondrial genomes are available at GenBank: MF352145–MF352171 and KJ493406MF.
