# Supplementary figures and images for: Increasing 28 mitogenomes of Ephemeroptera, Odonata and Plecoptera support the Chiastomyaria hypothesis with three different outgroup combinations
Source: PeerJ. 2021 Jun 22;9:e11402. doi: 10.7717/peerj.11402 (PMC8231340; doi:10.7717/peerj.11402)

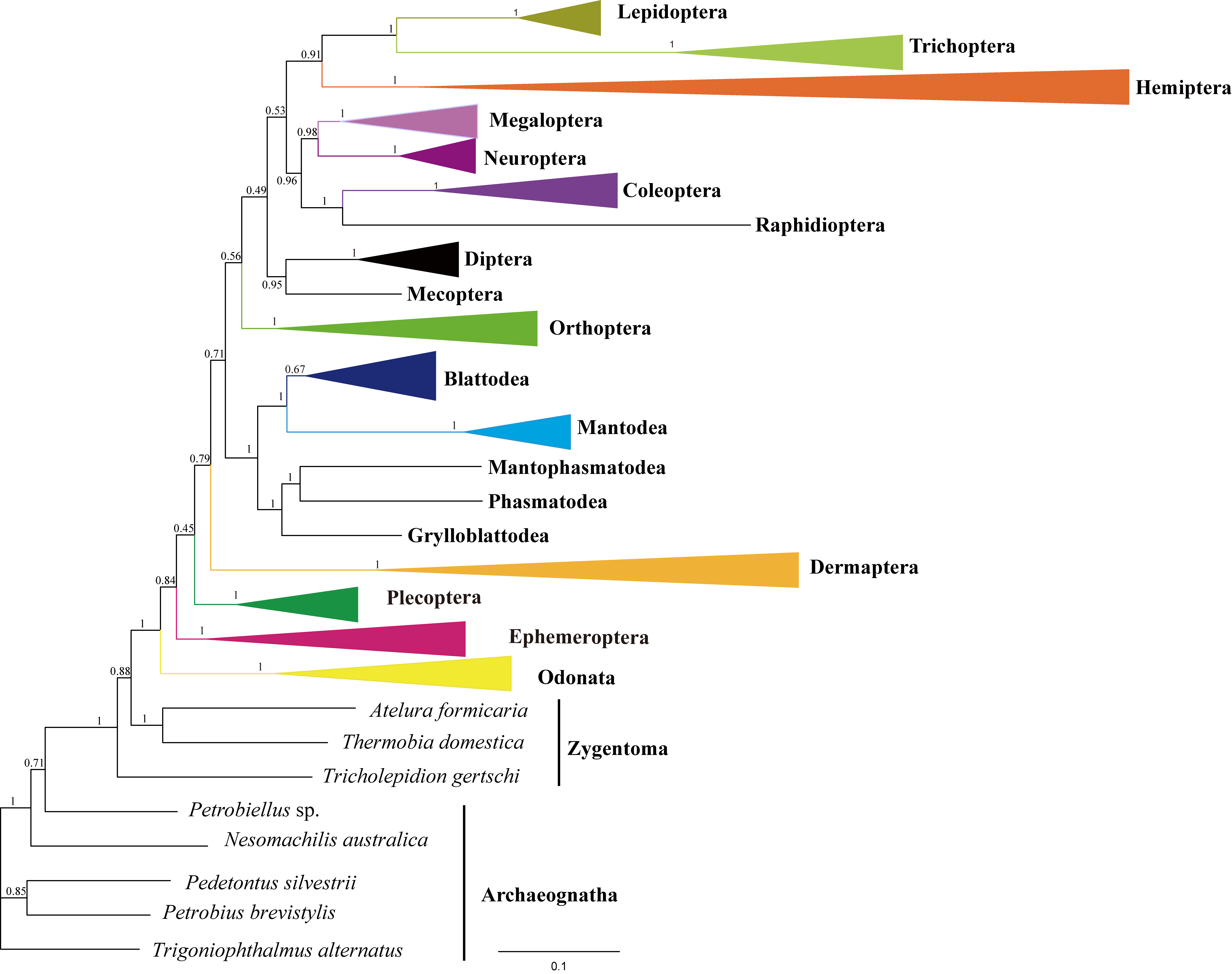

Supplement: Supplemental Information 1 — Phylogenetic analyses using nucleotide data were carried out for the 114 insect species based on all 12 protein-coding genes from their respective mt genomes. Five bristletails (Pedetontus silvestri, Petrobius brevistylis, Petrobiellus puerensis, Trigoniophthalmus alternatus, Nesomachilis australica) and three silverfishes (Atelura formicaria, Tricholepidion gertschi, Thermobia domestic) were used as outgroups. Numbers above the nodes are the posterior probabilities of BI. Subtrees of the monophyly of an Order were collapsed whereas the relationship within the Order is the same as Fig. 3. [file peerj-09-11402-s001.png]

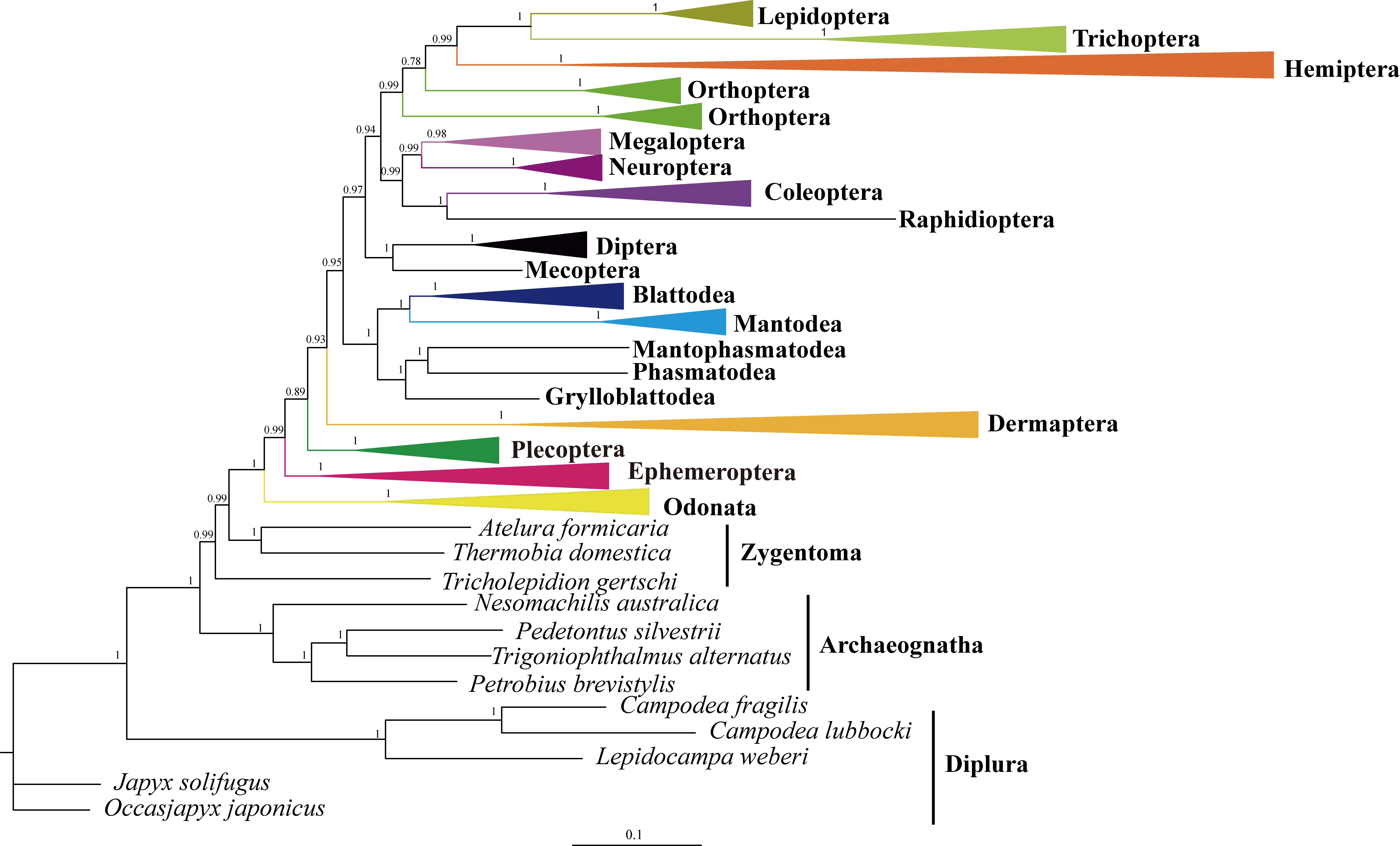

Supplement: Supplemental Information 2 — Phylogenetic analyses using nucleotide data were carried out for the 119 insect species based on all 12 protein-coding genes from their respective mt genomes. Five diplurans (Campodea lubbocki, C. fragilis, Japyx solifugus, Lepidocampa weberi, and Occasjapyx japonicus) were used as outgroups. Numbers above the nodes are the posterior probabilities of BI. Subtrees of the monophyly of the Order collapsed whereas the relationship within Order is the same as Fig. 3. [file peerj-09-11402-s002.png]

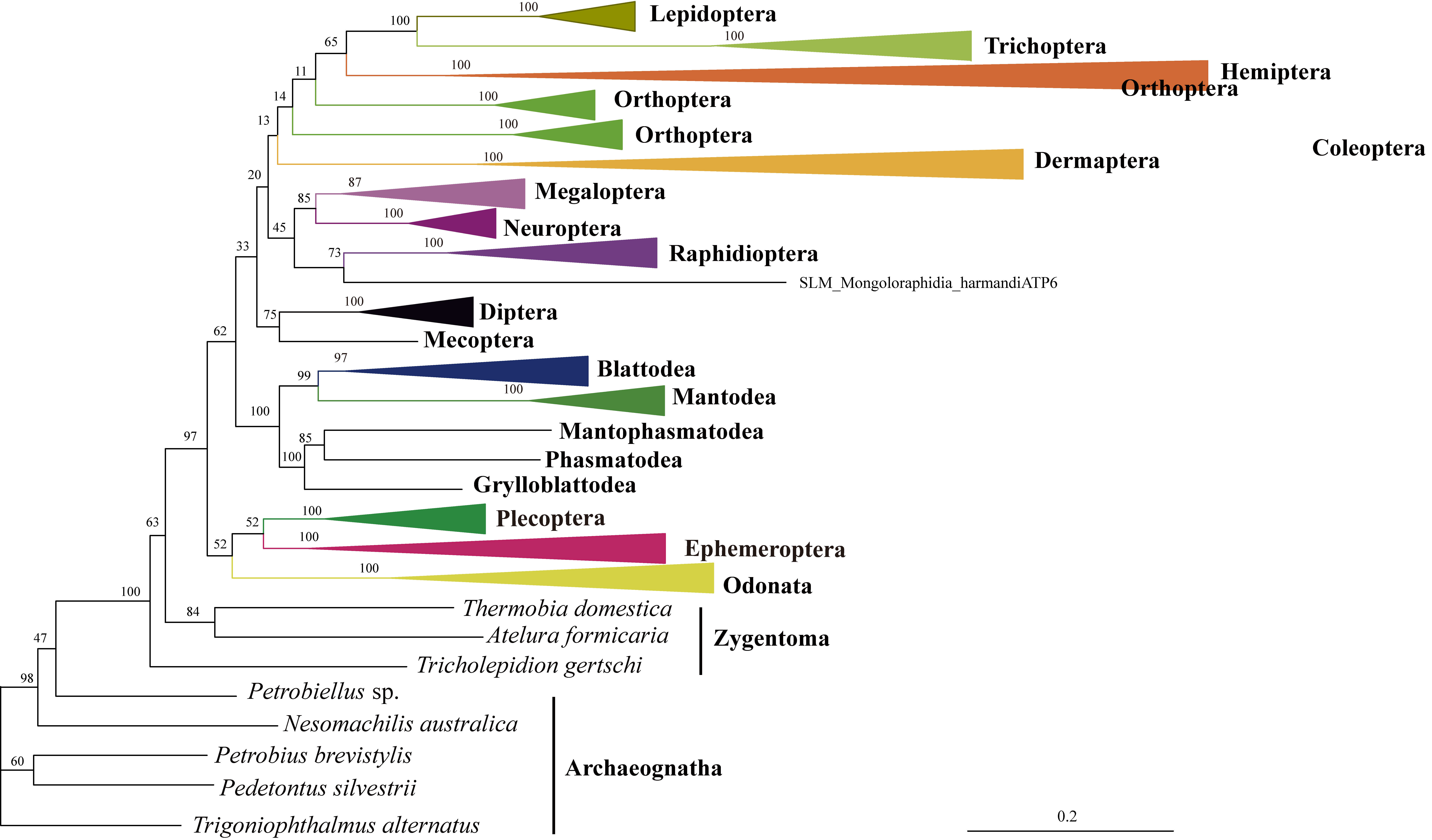

Supplement: Supplemental Information 3 — Phylogenetic analyses using nucleotide data were carried out for the 114 insect species based on all 12 protein-coding genes from their respective mt genomes. Five bristletails (Pedetontus silvestri, Petrobius brevistylis, Petrobiellus puerensis, Trigoniophthalmus alternatus, Nesomachilis australica) and three silverfishes (Atelura formicaria, Tricholepidion gertschi, Thermobia domestic) were used as outgroups. Numbers above the nodes are the bootstrap values of ML. Subtrees of the monophyly of the Order collapsed whereas the relationship within the Order is the same as Fig. 3. [file peerj-09-11402-s003.png]

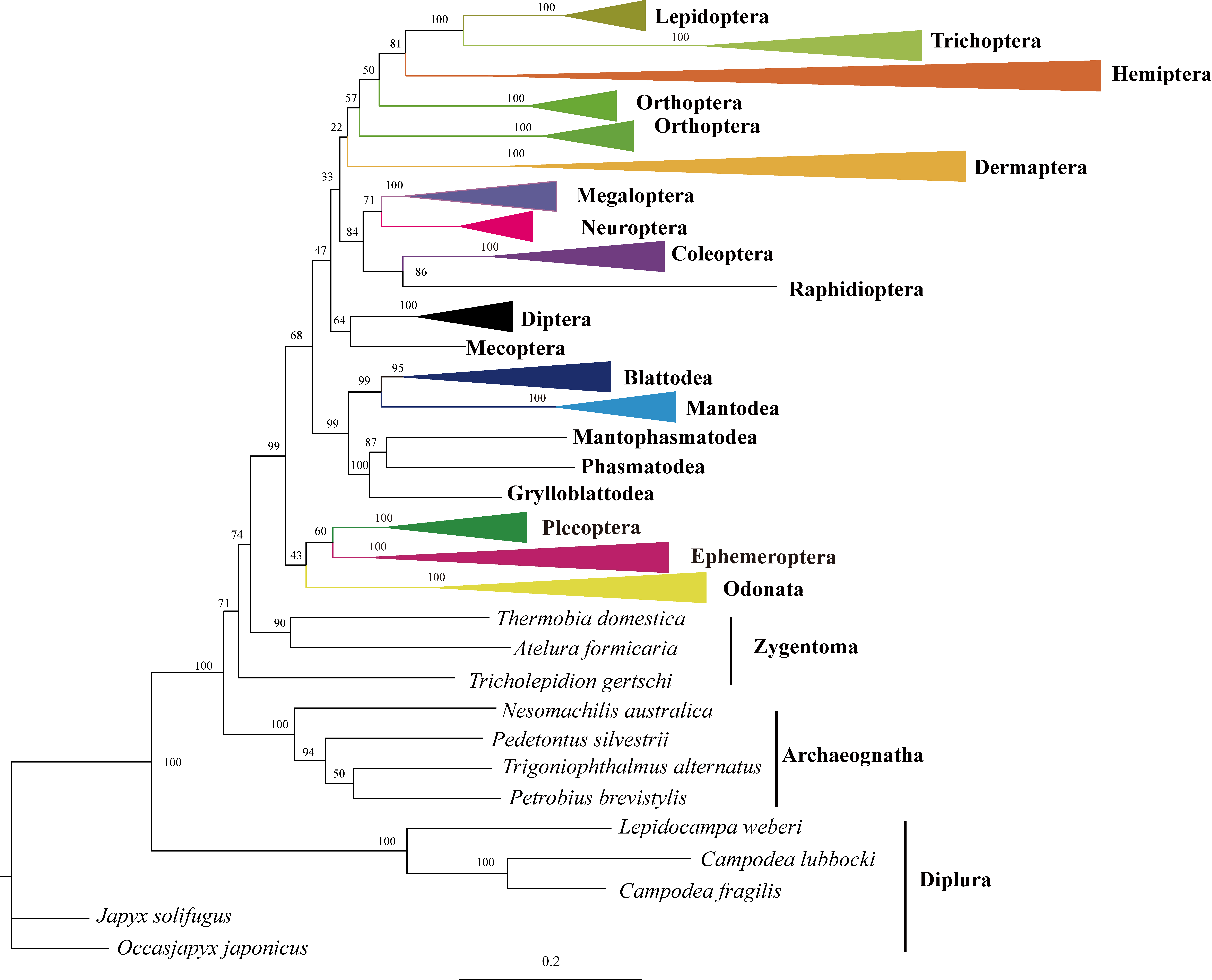

Supplement: Supplemental Information 4 — Phylogenetic analyses using nucleotide data were carried out for the 119 insect species based on all 12 protein-coding genes from their respective mt genomes. Five diplurans (Campodea lubbocki, C. fragilis, Japyx solifugus, Lepidocampa weberi, and Occasjapyx japonicus) were used as outgroups. Numbers above the nodes are the bootstrap values of ML. Subtrees of the monophyly of the Order collapsed whereas the relationship within Order is the same as Fig. 3. [file peerj-09-11402-s004.png]
